# Abstract of the Proceedings of the Buffalo Medical Association

**Published:** 1857-05

**Authors:** Sanford B. Hunt


ART. II.—*Abstract of the Proceedings of the Buffalo Medical Association.*

TUESDAY EVENING, April 7, 1857.

The association met.

Present—The President, Dr. Eastman, in the chair. Drs. Dayton, Mixer, White, Hutchins, Wilcox, Nichols, Treat, Whitney, Strong, Newman, Flint, Wyckoff, Hunt, Baker, Rochester, Lemon, Gay, Ring, Boardman and Underhill.

The minutes of the preceding meeting were read.

*Prof. Rochester* wished to have tenderness of the larynx on pressure included as one of the symptoms in the cases of croup reported by him at the meeting of last month.

On motion, Drs. Austin Flint, Jr., Sylvester J. Rankin, —— Cole, were invited to sit with the association until such time as they could perfect their claims to membership.

This being the annual meeting, the Tieasurer read a report of the official acts of the Trustees for the past year, which was accepted and adopted. The report announceed the lease of new and more commodious rooms.

The Secretary then presented a book containing the transactions of the association for the two years ending April, 1856, in a neatly bound and printed form. This was accepted wi:h a vote of thanks.

A ballot was then had for the officers and trustees for the ensuing year, with the following result:

| | | |
|---|---|---|
| AUSTIN FLINT | was elected | President. |
| C. C. WYCKOFF, | " | Vice-president. |
| SANFORD B. HUNT, | " | Secretary. |
| JAMES M. NEWMAN, | " | Treasurer. |
| B. H. LEMON, | " | Librarian. |
| C. L. DAYTON,<br>J. BOARDMAN,<br>A. W. NICHOLS, | } | Primary Board. |

On motion, the President named Drs. White, Mixer, and Gray, to conduct the officers elect to their chairs.

Prof. Flint, on assuming the duties of President, addressed the association. He said that he should make no set speech, but it was right to presume that his feelings would dictate some remarks. The association had now existed for twelve years. He had been intimately associated with the movement which gave it origin, had served with zeal for twelve years in its ranks, and was now for the first time the recipient of its honors. On looking back over its history, he could see that it had been individually and generally of great utility; and that it had now attained to a higher importance than ever before. By meeting and comparing ideas with each other, much useful information was conveyed, and each member profited by it. Again, the association was useful to the profession at large, by increasing the respect of the public for it. The mere fact that we met together to confer on the means of advancing our usefulness, was a credit to us with the public, and still more beneficial was the effect which it produced on the professional mind. Our meetings promote kindly feeling among us. The great cause of professional rivalries is the want of contact with each other, and as evidence of this benifit of association, he could point to the fraternal feeling now existing among us..

We have a right to infer that those who hold themselves aloof from associated movements for improvement, and isolate themselves from professional connections, have the cause of science and of fraternity but little at heart, or they would manifest it by some contributions to the general stock.

In concluding, Prof. Flint tendered his cordial thanks to his fellow-members for the high honor they had conferred upon him, and pledged himself to the performance of the duties of his office with zeal and impartiality.

*Prof. White* then moved

"That a vote of thanks be tendered to the retiring President, Dr. Eastman, for the able, dignified, and courteous manner in which he had performed the duties of his office. Carried.

A vote of thanks was also passed to the other officers.

It was here moved to suspend the usual order of proceedings. Carried.

*Dr. Newman* moved that in furtherance of the notice given by Prof. Flint, at the last meeting, to amend the by-laws so that the last sentence of section 1, chapter I, of the by-laws should read: "Every member shall also annually pay the sum of *three* dollars into the Treasury of the Association." Carried.

*Dr. Newman* gave notice that at the next meeting he should move to amend the by-laws, by striking out the words "two dollars on" in the last sentence of chapter II, section 5 of the by-laws.

Laid over under the rule.

*Prof. Flint* then read the following report on epidemic pharyngitis:

*Analysis of Twenty-three Cases of an Epidemic Fever characterized by mild, Erythematic Pharyngitis; with reference to the question of its identity with Scarlatina.* Communicated to the Buffalo Medical Association. By Austin Flint, M. D.

During the latter part of January and the early part of February last, I met, in private practice, with several cases of fever accompanied by mild pharyngitis and ending in convalescence, after a career of from three to five days. At first, in several of these cases, I supposed that the disease would prove to be scarlatina, which then, as well as before and since, prevailed to some extent in the city. The recurrence of cases bearing a close resemblance to each other, and the eruption of scarlatina being uniformly wanting, at length led me to expect the absence of the latter characteristic in the cases which I continued to meet with for several weeks. The question then arose, as it will now arise, whether this fever is to be considered as a variety of scarlatina or a distinct species of fever. Deeming this a question of interest and importance, I soon began to keep brief records of the cases that came under my observation, noting from recollection the facts pertaining to the cases already observed. I have collected notes of twenty-three cases which are nearly, if not quite all that have occurred in my practice* These cases I propose to analyze with reference mainly to the bearing of the results on the question just stated. In recording the cases I did not take pains to note all the symptoms with minuteness. I was satisfied to commit to my book of records a concise general statement of the clinical history of each case. For the present object my notes are sufficiently comprehensive. I shall proceed to present the analytical results; afterward I shall devote a little space to the inquiry concerning the identity or non-identity of the disease with scarlatina. In making the analysis I have distributed the historical facts into the following sections:

1. Age, sex, date, duration.
2. Degree and duration of febrile movement.
3. Affection of the fauces.
4. Symptoms referable to the skin.
5. Symptoms referable to the digestive system.
6. Symptoms referable to the urinary system.
7. Symptoms referable to the pulmonary system.
8. Symptoms referable to the nervous system.

---

* Several case have fallen under my observation since this report was written.

9. Miscellaneous symptoms.

10. Number of persons affected in the same family.

11. Sequels.

12. Preöccurrence of scarlatina in the persons affected, and the occurrence of scarlatina in the family or neighborhood previously or subsequently.

1. *Age, sex, date, duration.* In thirteen cases the persons affected were children, that is, between two and twelve years of age. In eight cases the patients were adults, and in two cases infants under one year of age. Of the eight adults, one was over sixty, another was fifty, another forty; two were thirty-five, and the remaining three were under thirty. Of the children the great majority were over five years of age.

Of the eight adults, five were males, and three females. Of the thirteen children, six were females, and seven males.

The first case occurred January 18th, and the second on the 25th of the month. The last case occurred on the 15th of March. Seventeen cases occurred in the month of February, and four in the month of March.

The duration of the disease was from three to six days. Exclusive of the infants, in every case the severity of the disease was sufficient to cause the patients to keep the bed at least for one day. In most instances they were too ill to sit up for two or three days. Generally they were convalescent on the fourth or fifth day. In one case only did the career of the disease extend to the sixth day. The termination of the disease was uniformly well defined by the cessation of febrile movement, disappearance of malaise and return of appetite, with the disposition and ability to sit up.

As regards the points embraced in this section, it is perceived that the disease evinced no marked predilection for either sex, nor for any particular period of life. Its epidemical character is shown by its prevalence during a limited period, viz: about two months. I may here remark that from conversing with my medical friends in this city, and also in the vicinity, I have learned that the affection, during the period named, was not confined to my own practice. I have reason to suppose that it prevailed considerably in this city and a considerable portion of the country. Dr. Strong of Westfield, Dr. Brandis of Erie, Dr. Campbell of Niagara, Dr. Mack of St. Catherines, Dr. Rogers of Suspension Bridge, and others, have informed me that cases similar to those which I have collected have fallen under their observation. Of the extent to which it has prevailed in this city, the reports, verbal or written, of the members of our association will enable us to form an idea.

2. *Febrile movement, its degree and duration.* In no instance was the febrile moment slight, but in all it was either considerable or marked. It continued for a period varying in the different cases from twenty-four hours to five or six days. In some of the cases a distinct exacerbation at evening was observed. The febrile movement in a few instances was ushered in by a chill or chilly sensations, but in no instance by rigor. Convalescence was established in all the cases shortly after the cessation of the febrile movement.

3. *Affection of the fauces.* More or less redness of the fauces was uniformly present. The redness varied in degree in the different cases. It wa marked or considerable in some, but in the greater number it was moderate or slight. In no instance did it present a dark, livid aspect. The affection was generally purely erythematic; moderate swelling of the tonsils was observed in two cases only. In the majority of the cases no exudation on the reddened membrane was apparent. In a few (six) small discrete patches were observed, pultaceous and speedily thrown off. Soreness of the throat was in no instance a prominent symptom; it was not generally a subject of complaint, nor was it manifest in the act of deglutition. The lymphatic glands of the neck were slightly swelled in most, perhaps in all instances; but, except in one case, not sufficiently to be apparent to the eye. In the excepted case just referred to, the enlargement disappeared without suppuration.

4. *Symptoms referable to the skin.* There was no eruption in any case save that in one patches of erythema, on the third and fourth days, appeared on the face and neck. In several instances, shortly after the attack, the skin presented an appearance of capillary congestion, as if the scarlatinous eruption was about to be developed. The occurrence of sweating is noted in two cases only.

5. *Symptoms referable to the digestive system.* Loss of appetite was a symptom in all the cases, continuing till the febrile movement ceased, after which the desire for food was gradually developed. Vomiting is noted but in one case. Thirst was not generally a prominent symptom. Looseness of the bowels existed in three cases. In the remainder of the cases there was more or less constipation.

The tongue was generally thinly furred. The strawberry tongue of scarlatina was not present in any case. Although not noted, I recollect observing in some of the cases the red points caused by the projection of the extremities of the papillæ above a thin coating; an appearance, however, frequently presented, in children especially, in various diseases. It is a fact important to be noted, that vomiting at the commencement of the disease, (a symptom so frequently present in scarlatina,) occurred in a single case only.

6. *Symptoms referable to the urinary system.* Nothing is noted under this head except scanty secretion of urine in one case. Attention to the urine as regards quantity even was not generally given, and in no case was the urine subjected either to chemical or microscopical examination.

7. *Symptoms referable to the pulmonary system.* In the great majority of the cases cough was not present. It is noted in the histories of five cases only. In all these instances it was dry, irritable, being evidently due to the pharyngeal affection. The attack was not preceded directly, nor accompanied, nor followed by bronchitis or any other pulmonary affection, in a single instance; nor was the affection of the throat preceded, accompanied or followed by irritation or inflammation of the greidinar membrane. The local complication was limited to the fauces.

8. *Symptoms referable to the nervous system.* The only symptoms falling in this section are as follows: In one case, at the commencement, palpitations occurred. The patient had formerly been subject to palpitations. In another case, (a female, aged 50) slight mental aberration was observed during the heighth of the febrile movement. In another case convulsions occurred on the first day, lasting for a few moments, and they continued to return, at intervals of two and four hours, during the space of twenty four hours. The patient, a child seven months old, had, in all, nine paroxysms of convulsions. They were apparently interrupted by the inhalation of chloroform, the child being kept quiet under the influence of this remedy for twelve hours. In another case, the head, shortly after the attack, was drawn far backward as in opisthotonos, and was constantly in that position for five days, gradually resuming its normal relation to the trunk after the cessation of the febrile movement, and other evidence of convalescence.

9. *Miscellaneous phenomena.* The histories contain nothing to be embraced under this head, except that in two cases there occurred a small discharge of purulent matter from the ears—'gatherings' succeeding ear-ache, so common in childhood.

10. *Number of persons affected in the same family.* Thirteen of the cases occurred in five families; each of the remaining cases were in a distinct family. Of the thirteen cases just mentioned, two were fellow boarders, but not room-mates; four were in one family, viz: two children, a grandmother and an aunt; two were children of the same parent; three were a mother and two daughters, and two were a brother and sister.

The cases in the same family occurred, in the relation of time, as follows: 1st. The two fellow boarders were attacked successively, after an interval of a week. 2d. The four members in the following order: first, the aunt; three weeks afterward one of the children; about the same time the grandmother, and after eleven days the other child. 3d. The two children, successively, after an interval of thirty days. 4th. The mother and two daughters in the following order: first, a daughter; twelve days afterward the other daughter, and seven days afterward the mother. 5th. The brother and sister were successively affected after an interval of three weeks.

11. *Sequels.* One of the patients after some weeks had inflammatory croup. One had diarrhœa and one gathering in the ear. These are all the sequels that have occurred. More than a fortnight has elapsed since the last of the cases was observed. The majority of the cases occurred from four to six weeks prior to the time I am writing. General dropsy or œdema has not taken place in any instance. There has been no evidence of disorder of the urine in any case, nor has desquamation of the cuticle occurred in any single instance.

In general, the system was left by the disease considerably debilitated, the patients recovering their usual strength slowly, but convalescence not retarded by any appreciable, newly developed events.

12. *Reäppearance of scarlatina in the persons affected, and the occurrence of scarlatina in the neighborhood previously or subsequently.* In eight of the cases the preöccurrence of scarlatina was either unknown or not ascertained. Of the remaining fifteen cases, in eleven the patients had never had scarlatina, and in four it was known that this disease had been previously experienced. In three of the latter cases I had attended the patients during scarlatina.

In not one of the twenty-three cases had a case of scarlatina occurred in the family, nor did a case of scarlatina follow in a single instance. In this statement it is of course assumed that the cases which have been now analyzed were not cases of scarlatina.

I may add to the above statement, that in no instance was it known that there had been any exposure to the contagion of scarlatina.

From the foregoing results of the analysis of the histories of the cases which I have collected, we are justified, as I think, in the following deductions: This disease was an epidemic fever characterized by mild, erythematic inflammation of the fauces as a constant local complication. Its character as essentially a fever is established by the febrile movement being in so marked a degree out of proportion to the local affection; in other words, evidently not being symptomatic of the latter, and by its running a definite although a brief career. It is a fever of from three to five days' duration. Its epidemic character is sufficiently apparent. It has prevailed more or less extensively in this city for about two months, reaching its acme gradually, declining gradually, and at length nearly disappearing, affecting both sexes and different ages without notable discrimination.

As an epidemic fever its symptomatic features were very uniform. The erythematous affection of the fauces constitutes the only positive character, aside from the brief duration of the febrile career. The other symptoms uniformly present were only those incident to febrile movement; and the symptoms observed in a few cases, viz: the convulsions in one case, the retraction of the head in one case, etc., were only incidental events not intrinsic elements of the disease.

The small patches of white exudation observed in some of the cases, do not suffice to establish any relation of the local affection to that called *diphtheritic*, as described as Bretenneau and others.

The occurrence of several cases repeatedly in the same family does not suffice to prove that the disease was propagated by contagion, since this fact is explicable on the supposition of the patients being equally exposed to an epidemic influence, and there being a marked discrepancy in the intervals separating the cases successively occurring in the same family.

We now come to the question, was the disease a variety of scarlatina, or is it to be regarded as a distinct species of fever?

An essential distinction between two diseases involving a special causation, is a difference in the cause of each such as renders it impossible for that of the one under any circumstances to give rise to the other. The distinction between different species of diseases, in other words, is the same as between different species of plants or animals; each species springs from a separate, independent germinal source. Two essentially distinct desires are never mutually convertible; but this is not denying that they may coëxist in the system at the same time, and give rise to a mixed or hybrid affection.

The special causes of disease, especially of epidemic diseases, are unknown, and hence, in determining differences of species among affections which resemble each other more or less, we must study and compare, respectively, the laws regulating their origin, development and progress, together with their symptomatic phenomena and lesions. In fact, owing to our limited knowledge of etiology and pathology, we are obliged to infer an essential difference in special causes from an investigation of their effects, which effects are the historical facts pertaining to the diseases to which the causes give rise.

Now, do the laws of the origin, development and progress of the disease in question, with its symptomatic phenomena, so far as they may be predicated on the results of the foregoing analytical investigation, show the disease to be due to the special cause which produces scarlatina—scarlatinous poison—or must we assume that it has its own, separate special cause? Without asserting that the data before us are sufficient to warrant a conclusion as positive as might be desired, the following considerations may be adduced in support of the opinion that this disease is specifically distinct from scarlatina:

1. The absence of the scarlatinous eruption. The occurrence of cases of *scarlatina sine eruptione* is not to be doubted. Exclusive of the instances in which death takes place prior to the eruptive stage, the eruption is wanting in certain cases in which the affection of the throat is unusually severe—the local manifestation of the disease being concentrated on the fauces. Assuming the cases which I have analyzed to be cases of scarlatina, this explanation of the absence of the eruption is not admissible, since in all, the throat affection was mild. But we meet with instances, during the prevalence of scarlatina, of mild pharyngitis, with more or less febrile movement and without an eruption, which are probably due to the scarlatinous poison. The mildness of the disease in these cases, it may be presumed, accounts for the absence of the eruption. This explanation is hardly applicable to so large a series of cases, in many of which the febrile movement was quite intense, and the severity of the disease in most instances sufficient to cause the patients to keep the bed for one, two or three days.*

2. The unform absence of any connection with well marked cases of scarlatina occurring either previously or subsequently. In the exceptional instances of *scarlatina sine eruptione*, the disease is generally referable to

* I have recently met with several cases of scarlatina, in which, with a copious eruption and a greater degree of pharyngitis, the febrile movement and general disturbance of the system, were much less than in the milder cases of the epidemic described.

known exposure to the scarlatinous poison. These instances occur among persons who are brought into contact with patients affected with scarlatina. Whether in these instances a contagious miasm is reproduced is a matter of question. Now, it is a striking fact with respect to the cases which have been analyzed, that in no one instance had the patients been known to have been in contact with persons affected with well marked scarlatina. In no instance was the disease preceded or followed by scarlatina in the same family. To suppose that in twenty-three consecutive cases the disease could not be traced to some exposure to contagion, if it were a variety of scarlatina, is not consistent with medical experience.

3. Scarlatina shows a very marked predilection for young patients. Adults, and especially persons beyond middle life, are very rarely affected. Now, of the cases analyzed, several were cases in which adults and persons beyond middle life were affected.

4. None of the sequels of scarlatina occurred, viz: rheumatism, serous inflammation, and especially dropsy. Out of twenty-three consecutive cases of scarlatina, the latter sequel would be expected to occur in a greater or less number of instances. This sequel is more apt to follow mild than severe cases, and hence, assuming these to have been cases of a variety of scarlatina, the chances of the occurrence of this sequel would be not less, certainly, than in well marked cases of the disease.

5. In several instances the patients affected with the disease had had scarlatina, the proportion probably being much larger than of those who are affected a second time by the scarlatinous poison.

10. Assuming the disease to be specifically distinct from scarlatina, the question asises, where is its appropriate nosological place? or is it a species of fever as yet without a 'local habitation and a name?' I do not propose to discuss this question. The discussion would require too much space, and moreover, the necessary data are not readily available. Epidemic pharyngistis is not a new disease. The disease known by this title is probably essentially a fever with pharyngitis as a local characteristic. Presenting a diphtheritic exudation, and tending to extension into the larynx, it has prevailed at certain times and places with great fatality. Certain of the cases of epidemic erysipelas, vulgarly black tongue, are cases of fever with pharyngitis as the prominent or sole local complication. Finally, the cases of this disease bear a close analogy to cases of influenza, except that the local characteristics of the latter are seated in the sueiderian and bronchcial mucous membrane.

It may perhaps seem that I have given to this disease a more extended consideration than its importance claims. The main object of the investigation, however, viz: to determine the identity or non-identity of the disease with scarlatina, is important, as well as in a scientific point of view, interesting. To determine the different species of disease is certainly a great desideratum, and there is reason to believe that there is even now ample scope for the clinical observer and historian to prosecute studies with reference to this end.

MARCH 31, 1857.

*Prof. Rochester* then read the following report on the same subject:

At the February meeting, Prof. Flint called the attention of the Society to the prevalence of an epidemic form of pharyngeal disease. The writer of this communication was not present, and consequently had not the pleasure of hearing the remarks of that gentleman. He learns, moreover, from Prof. Flint, that it is his purpose to report at length the cases then and since observed; it is therefore by his permission that the following paper is presented to the Society as a contribution upon a subject which will be more fully and completely elaborated by him. From the 6th of January to the 4th of April, thirty-seven cases have been noted. The very general prevalence of scarlatina and influenza within this period, may have caused an error in diagnosis in a few cases, for that the latter affection should assume some of the pecularities of the former would be expected, and the blended or modified disease thus presented would not be unlike that termed pharyngitis. Care has been taken, however, in the cases enumerated, not to include any who have been directly exposed to scarlatina.

In all the cases observed by the writer, there has been a similarity of both local disease and general symptoms, varying, however, very much in intensity and duration, and ranging from limited infection, with scarcely appreciable constitutional disturbance, to severe angina with febrile movemovement and general malaise and prostration. The attack has usually been sudden, consisting, in a majority of cases, of febrile movement, not intense, however, with slightly accelerated but feeble pulse. In every case the tongue has been furred, with the papillae more or less prominent. In almost every instance there has been complete loss of appetite and a more than corresponding sense of debility. The local disease has often been so slight as not to have attracted the attention of the patient; but even in the mildest cases, inspection has revealed either a diffuse or punctate redness in the fauces, and notably about the outer margins of one or both palatine arches. The constitutional disturbance has always been greater than the local disease would seem to warrant, for the latter has in no instance been of such magnitude or intensity as properly to be termed grave. In but two instances has tonsillitis, and that slight, supervened; in four there has been an œdematous condition of the uvula. Deglutition has not been painful or difficult in any case, but *inappetence* has been a characteristic condition. Spontaneous vomiting has occurred frequently with children, and occasionally with adults. Cough has usually attended the affection, at times assuming a laryngeal or croupy character; in a few cases, and especially at night, it has been very troublesome. With but one exception it has not depended upon any appreciable pulmonary complication, and as would be inferred, there has been but slight expectoration. I can find no substitute expression of the manner of the matters ejected, equivalent to the French *exscréation.*

The febrile movement has in some been tolerably constant, but in a large majority of the cases it has been decidedly intermittent, but intermittent without distinct periodicity. Two exacerbations have been often noticed in one day, and the rule (subject to exceptions) has been an increase of febrile movement on alternate days, but not at corresponding hours. Another peculiarity of the same character, is the uncertainty of the establishment of convalescence. In many patients, evidently improving, the disease has suddenly returned, as manifested both by general symptoms and the reäppearance in force of the local affection, or its invasion of parts hitherto intact. The subjects of the attack, as observed in the cases under consideration, have been of all ages and conditions. The severest examples were presented in a gentleman of delicate constitution, 58 years of age, and in a robust infant 20 months old. The former was confined to his bed for seven days, and to his room a fortnight longer. The latter was extremely ill for four weeks, and convalescence was not fairly established until the expiration of the seventh. This case may have been complicated with lobular pueumonia, but if so it was not detected after repeated careful explorations. The child was seen in consultation by Prof. White, at the request of the writer.

On three occasions during its illness, diarrhœa set in spontaneously, and continued for several days. This is mentioned by Valleix, Guersant and others as a not infrequent accompaniment. The oldest person attacked was a female 62 years of age; the youngest an infant of four months. The average duration of the disease was seven and a half days—that is, counting from the cessation of the febrile action, and the subsidence of the pharyngeal inflammation; the period would be much greater if it were extended to the time of cessation of debility, cleaning up of the tongue, and restoration of appetite; the cough also is often maintained for a considerable time beyond that specified.

The present epidemic of pharyngitis does not correspond, according to the writer's observation, precisely with any of the forms described by Valleix and other writers quoted by him. It approaches more nearly to that termed *gutturale*, (being, however, more severe,) than to the "*pharyngite couenneuse ou dipthérite*," a very much graver affection. Both of these are stated to assume an epidemic character, and are regarded as distinct from the "*pharyngite pultacée*," which is directly associated with scarlatina. A few of the cases seen by the writer might be termed diptheritic, if that is applicable in this connection, to the presence of superficial ulcers, covered by a slight film, appearing upon the mucous membrane of the lips and buccal cavity after the cessation of the febrile movement. As these occurred only in a few subjects, and those strumous children, the ulcers were not thought to be necessarily connected with the coëxisting or preëxisting pharyngeal disease.

*Propagation of the Affection.* An epidemic influence being admitted, it is very difficult to determine whether the disorder is or is not directly communicable from individual to individual. The probability of its communicability has, however, repeatedly occurred to the writer. Whole families have been attacked, the several members not simultaneously, but in succession, while servants and others not occupied in attendance upon the sick have escaped. This statement does not apply universally, but there have been but few exceptions. The period of incubation is short, the disease manifesting itself in some instances within twenty-four hours after exposure. It resembles in this respect the *pharyngite couenneuse* described by Valleix. Many of those attacked had previously (an interval of one or more years having elapsed,) had scarlet fever; the majority, however, had never had that affection. Two children who were very ill with scarlatina, had pharyngitis with marked febrile action, one month after their recovery from the former. This would seem to demonstrate that the two affections had nothing in common.

*Treatment.* Evacuants have not been employed to any extent; advantage in some instances has followed the use of an ipecacuanha emetic. Dovers powder in small doses, singly or combined with calomel or James' powder, has appeared to be of great benefit. Its efficacy has certainly been increased in many cases by the mercurial combination. Quinine has been found very useful, not only as a tonic, but as a febrifuge and anti-periodic, and still further, as exercising a special control over the morbid action of the mucous membrane. It has been employed in almost every case, and nearly always with advantage; certainly never with disadvantage.

*Local Treatment.* This has consisted of the inhalation of the vapor of warm water, the use of emollient and of stimulating gargles, and of the topical application of nitrate of silver, (grs. x to xxx to ℥ j.) The solution of nitrate of silver is the only one of the direct applications from which positive advantage was derived. It was only used in six cases, and all made an early and rapid convalescence. It was not employed more frequently, on account of the tender age of many of the patients, and because the nature of the malady was not so grave as to require so disagreeable a remedy. In one patient it controlled both cough and vomiting, where anodynes and alteratives failed to do so.

With the permission of the Society I will now report as addenda to the above, two cases of fatal disease, in which pharyngitis was found as a complication:

CASE I. Robert B., aged six months. March 21st. My attention was accidentally directed to this child by its mother, upon whom I had called to leave a message for its father. Its symptoms were like those presented in the advanced stages of pseudo-membranous croup, and yet strangely enough its parents had not apprehended any danger. It died on the succeeding day. Its history, as far as ascertained, was as follows: Although a well grown and fleshy child, it had never been healthy; from birth it had presented a squamous, dark colored eruption upon its back, and an erythematous one about the ears, groin and nates. One month preceding its death it had taken a severe cold, and never afterwards cried *articulately*, although when in pain, tears were often seen on its cheeks. It always nursed vigorously, until the last two days of its life. Although unable to cry aloud, its cough was hoarse and audible, and especially so on the night preceding its death. During the last three days of its illness, the erythmatous localities referred to above augmented very rapidly, and displayed a thin white film in spots. Was this the *diptherite cutanee* of the French writers?

*Autopsy eighteen hours post-mortem.* This was conducted by Dr. A. W. Nichols, who had also visited the patient with me several times. Rigor mortis excessive, head not examined; organs of thorax and abdomen healthy, save a solitary tubercle in the apex of left lung; pharynx, the base of the tongue and the whole pharyngeal membrane was studded with minute pearl-colored elevations of a cyst like character; these, when opened, discharged a minute quantity of milky serum; they were evidently of an exudative character; the exudate was firmly attached, and appeared to dip down into the mucous crypts; trachea, opened on its posterior surface, the whole living membrane was injected, reddened and tumified; it was covered with a considerable coat of glairy tenaceous mucus; on the middle anterior line, between the superior and inferior *vocal cords*, and eroding the margins of both, *an ulcer of irregular oval shape*, a line and a half in diameter, was seated; it had entirely destroyed the subjacent mucus membrane; the specimen is presented to the society for inspection, as also one of more marked and purely pharyngeal disease, to which I shall briefly refer. The ulcer and the lining membrane of the trachea explain the croupy symptoms. Did the pharyngitis produce the ulcer? This does not appear from the history of the case; the ulcer had probably existed for nearly a month, dating from the time of the observed extinction of voice. The appearance of the squamous dark colored eruption upon the back, which was congenital, points also towards a possible specific origin. We cannot fix the date of the pharyngitis, and consequently it is impossible to say that it had any connection with the tracheal ulcer. It is the impression of the writer, however, that the primary affection was pharyngeal, and that it extended to the trachea.

Case II. William H., mulatto, aged seven months. The history of this case is even more imperfect than the last. At 5½ A. M., April 4th, I was summoned in great haste, arriving at 6 A. M.; the child was dead. I had seen it six weeks before; it had then impetigo larvalis, otherwise in good health. I ordered cleanliness and cod liver oil; under this regimen it improved rapidly. About a week before its death it took a slight cold; on the evening of the 3d it was feverish and cross, and vomited the breast milk; at 3 A. M. on the 4th it cried and nursed; at 5 A. M. the mother woke, the child lay heavily upon her arm; it was in a cold sweat; her husband came for me; before he returned it was dead. Query. Did it not die in a convulsion? The upper incisors were nearly through the gums.

*Autopsy.* By Dr. A. W. Nichols; six hours post-mortem. Rigor not established; head not examined, the parents not consenting; organs of thorax and abdomen healthy; larynx and trachea healthy; œsophagus healthy; pharynx, the same condition as found in Case I., but as the parts have been recently removed, and have not been macerated, the enlarged mucous follicles, the injection of the mucous membrane, and the pearl-colored elevations containing the milky serum, can all be readily seen by the members of the society. Although not strictly the cause of death, may it not be inferred that the pharyngitis occurring while dentition was active, was the agent that gave rise to the fatal convulsion? It is to be further noted that an erythematous condition was observed in this case, as in the preceding, occupying either groin, and covered with the same white film.

*Prof. Rochester* then exhibited the two pathological specimens alluded to in the report.

*Dr. Boardman* stated that this epidemic had been prevailing among the children at the Orphan Asylum under his charge. Some fifteen or twenty cases had occurred without any deaths. There had been no scarlatina in the house, and as further evidence of the non-identity of the two diseases, he mentioned that one child suffering from it had had scarlatina some eight or nine weeks previously, and six weeks before entering the Asylum.

*Dr. Wyckoff* said that on January 3d he was called to a case which he supposed to be scarlatina, having the sore throat and other symptoms of that disease, but not the eruption. The child had been exposed. Thirteen days thereafter another child in the same family had scarlatina; ten days later a servant girl had it; ten days after that another child had it; on Feb. 12 the child first mentioned had it with a distinct eruption; March 4th the mother came down with the symptoms, but without the eruption; and finally, on March 10th, the father had it with eruption, and was quite sick. All recovered.

On April 10th the mother was confined and gave birth to a healthy child, with desquamation of the cuticle on its hands and feet, giving rise to the supposition that it had had scarlatina in utero.

*Prof. White* reported two cases of placenta prævia, both of special interest, not only for the variety of their complication of labor, but for the peculiar circumstnnces which attended them.

Case I. Mrs. L. S. W., aged 28 years, menstruated last on the 10th of November, 1856. This was her sixth pregnancy. Was called to her on the 31st of March, in consultation with Dr. Wilcox. Mrs. W. had flowed somewhat during the fourth month, and since that time had flowed increasingly; for the last ten days very considerably. During all this time there were occasional severe gushes of hæmorrhage. Saw her at 10 A. M. with Dr. Wilcox. She was then in a condition of syncope, the os undilated. A tampon was introduced, and firmly supported. The hæmorrhage being checked, Dr. White left the patient in charge of Dr. Wilcox, until 12 M., the tampon being in the meantime firmly supported in its place. At 12 noon no change. Quietude was ordered, the head depressed and a bandage applied, and Tr. Ergot. Aeth. with Tr. Ferri Muriatis, administered; pulse feeble. At 2 P. M. the pains increased and pushed away the tampon and a fœtus of four and a half months. The placenta was found attached to the os uteri, which was very tender. After removal it was found unusually large and carnified.

This early hæmorrhage is very unusual. Writers assign the period of obliteration of the neck—six months—as that when it comes on from placenta prævia. Cazeau maintains that it is due to unduly rapid growth of the placenta—a view which seems to be confirmed by this case. The symptoms here were characteristic of placenta prævia. The treatment was that dictated by the emergency, as no author has written upon placenta prævia at this stage of pregnancy. Turning was impossible, and the tampon seems the only remedy.

Case II. Ellen ——, married, aged 21 years, entered the Lying-In Hospital during the winter months, has had one slight hæmorrhagic discharge. Dr. Rankin, the interne, found the placental souffle in region of bladder, and made a diagnosis accordingly. For fifteen days there was slight hæmorrhage. Labor took place on the 30th of March, and was so rapid that it was concluded before Dr. Rankin arrived. Dr. R. found the placenta adherent to the os, on one side. The child was vigorous when born, but died subsequently. The case was instructive, as teaching the value of the second sound, and the possibility of safe delivery.

*Prof. White* also reported a case of puerperal fever. A lady, prima para, 28 years of age, was delivered by instruments. Two or three days after had a chill followed by tympany and abdominal tenderness, with a pulse of 160. She took morphine in grain doses every two hours from Friday to Monday, with lesser quantitities thereafter. The strength was supported by the usual means. There was no stool for seven or eight days. Owing to excitement she relapsed, when the same treatment was reïnstated, when she recovered.

*Prof. White* also reported some cases in which he had used the yeast poultice as an anæsthetic, in carbuncle and other open surfaces, with much relief to pain from the carbonic acid gas. He thought it would be applicable to any irritated surface, or in burns or scalds.

*Dr. Treat* moved that the reports of Professors Flint and Rochester be referred to those gentlemen as a committee, with a request to report further.

In the course of conversation, it was stated that the close resemblance and almost identity of ideas between the two reports of these gentlemen, might suggest the idea that they were prepared with reference to each other. It was stated by them that they had had only a casual and momentary conversation, and were not aware of the nature of each other's report.

And the association then adjourned.

SANFORD B. HUNT, Secretary.

